# Chest CT imaging characteristics of COVID-19 pneumonia in preschool children: a retrospective study

**DOI:** 10.1186/s12887-020-02140-7

**Published:** 2020-05-18

**Authors:** Yang Li, Jianghui Cao, Xiaolong Zhang, Guangzhi Liu, Xiaxia Wu, Baolin Wu

**Affiliations:** 1 Department of Radiology, Xiangyang No. 1 People’s Hospital, Hubei University of Medicine, Xiangyang, 441000 Hubei PR China; 2 Department of Radiology, Taihe Hospital, Hubei University of Medicine, Shiyan, 442000 Hubei PR China; 3 Department of Neurology, Xiangyang No. 1 People’s Hospital, Hubei University of Medicine, Xiangyang, 441000 Hubei PR China; 4 Huaxi MR Research Center (HMRRC), Functional and molecular imaging Key Laboratory of Sichuan Province, Department of Radiology, West China Hospital of Sichuan University, No. 37 Guo Xue Xiang, Chengdu, 610041 Sichuan PR China

**Keywords:** COVID-19, Pneumonia, SARS-CoV-2, Multidetector computed tomography, Child

## Abstract

**Background:**

Recently, the World Health Organization has declared the coronavirus disease 2019 (COVID-19) outbreak a public health emergency of international concern. So far, however, limited data are available for children. Therefore, we aimed to investigate the clinical and chest CT imaging characteristics of COVID-19 in preschool children.

**Methods:**

From January 26, 2020 to February 20, 2020, the clinical and initial chest CT imaging data of eight preschool children with laboratory-confirmed COVID-19 from two hospitals were retrospectively collected. The chest CT imaging characteristics, including the distribution, shape, and density of lesions, and the pleural effusion, pleural changes, and enlarged lymph nodes were evaluated.

**Results:**

Two cases (25%) were classified as mild type, and they showed no obvious abnormal CT findings or minimal pleural thickening on the right side. Five cases (62.5%) were classified as moderate type. Among these patients, one case showed consolidation located in the subpleural region of the right upper lobe, with thickening in the adjacent pleura; one case showed multiple consolidation and ground-glass opacities with blurry margins; one case displayed bronchial pneumonia-like changes in the left upper lobe; and two cases displayed asthmatic bronchitis-like changes. One case (12.5%) was classified as critical type and showed bronchial pneumonia-like changes in the bilateral lungs, presenting blurred and messy bilateral lung markings and multiple patchy shadows scattered along the lung markings with blurry margins.

**Conclusions:**

The chest CT findings of COVID-19 in preschool children are atypical and various. Accurate diagnosis requires a comprehensive evaluation of epidemiological, clinical, laboratory and CT imaging data.

## Background

As a novel coronavirus disease, the coronavirus disease 2019 (COVID-19) emerged in Wuhan, Hubei, China in late December 2019. It is a highly infective disease caused by the severe acute respiratory syndrome coronavirus 2 virus (SARS-CoV-2; previously provisionally named 2019 novel coronavirus or 2019-nCoV). Then, the disease spread rapidly in the other regions of China and even abroad, and has become global health emergency as declared by the World Health Organization [1]. As of March 17, 2020, the total number of COVID-19 confirmed cases in the world is close to 200,000. The outbreak of COVID-19 has seriously affected people’s daily life, physical and mental health, and economic activities. At present, researches on COVID-19 suggest that patients of all ages are susceptible to the disease, and individuals with comorbid conditions are more susceptible and tend to have poor outcomes [2].

Previous studies on clinical and chest CT imaging characteristics of COVID-19 are mainly focused on adults [2–6]. A recent report based on 1099 patients with laboratory-confirmed COVID-19 indicates that the most common symptom in adult patients is fever (43.8%) on admission, the percentage of which is more higher during hospitalization (88.7%) [7]. The typical CT imaging manifestation of COVID-19 pneumonia is multifocal bilateral ground-glass opacities (GGO) with patchy consolidation that tends to develop in the outer zones of the lung and particularly in the subpleural regions [3, 8–10]. Due to the immaturity of immune systems in children, more attention should be paid to those subjects in the prevention and control of COVID-19. However, limited data are available for children with COVID-19 pneumonia. As a simple and quick imaging tool, CT can accurately detect and assess the lung lesions pediatric patients with COVID-19 pneumonia. To date, CT findings have been recommended as major evidence for clinical diagnosis of COVID-19 in Hubei, China. In the present study, we aimed to conduct a retrospective study to investigate the clinical and chest CT imaging characteristics of COVID-19 in preschool children.

## Methods

### Study population

The Institutional Ethics Committees of the Xiangyang No. 1 People’s Hospital and Taihe Hospital approved this retrospective study and waived the requirement for patient informed consent. From January 26, 2020 to February 20, 2020, eight preschool children with laboratory-confirmed COVID-19 from the Xiangyang No. 1 People’s Hospital, Hubei University of Medicine, Xiangyang, Hubei, China and Taihe Hospital, Hubei University of Medicine, Shiyan, Hubei, China were enrolled in the present study. These patients have not been included in any other reports, and the datasets used in the current study are available from the corresponding author on reasonable request. The inclusion criteria were 1) patients aged less than 6 years; 2) patients with complete clinical history information and CT image data; and 3) patients with good chest CT image quality. The diagnosis of COVID-19 was based on the *Guidelines for the Diagnosis and Treatment of Novel Coronavirus Pneumonia (6th Trial Edition)* proposed by the National Health Commission of the People’s Republic of China [11]. All patients were positive for COVID-19 after detection of SARS-CoV-2 from nasal swabs or aspirates via laboratory testing with real-time reverse transcriptase polymerase chain reaction (RT-PCR). The CT images and clinical data of all patients were collected. All patients were administered with anti-viral and supportive treatment, and prevention of complications based on their clinical condition.

### Acquisition of chest CT images

Plain CT scans were performed for all patients using multi-detector CT scanner (Toshiba, Tokyo, Japan; Emotion 16, Siemens, Erlangen, Germany) at the time of admission. Patients were placed in a supine position with their breath held at the end of inspiration, and the scanning range was from the chest entrance to the bottom of the lungs. The parameters used were as follows: thickness of the slices 1 mm, interslice gap 1 mm, matrix 512 mm × 512 mm; and tube voltage 80 kV, current 200 mA, pitch 0.813/HP 65.0, and dose-length product 36–51 mGy∙cm. The CT scan was performed by technicians with over 5 years of experience. Comprehensive protective measures for technicians included wearing isolation gowns, caps, masks, protective goggles, gloves, and shoe covers. Scanning rooms were regularly disinfected. Children under the age of 4 years were examined after sedation to reduce artifacts. All patients wore masks for protection.

### Chest CT image analysis

Initial CT images of the eight patients were viewed at the picture archiving and communication systems (PACS) workstation, with window settings optimized for the assessment of the lung parenchyma (width 1500 HU; level − 500 HU) and of the mediastinum and pleura (width 300 HU; level 40 HU). Two senior radiologists independently carefully observed and recorded the lung markings, the distribution, shape, density and number of the lung lesions, and other features that included the presence of hilar or mediastinal lymphadenopathy, and presence of pleural thickening or pleural effusion, based on the transverse view, combined with multi-planar reformation and other methods. A final determination was reached through consultation between the two radiologists. When the opinions were inconsistent, a third chest CT specialist with over 10 years of experience was brought in for discussion to make an agreement.

### Clinical typing

Clinical typing of patients with COVID-19 was evaluated according to the *Guidelines for the Diagnosis and Treatment of Novel Coronavirus Pneumonia (6th Trial Edition)* [11]. The severity of COVID-19 was classified into 4 categories: ① mild type: patients with mild clinical symptoms and no pulmonary changes on CT imaging; ② moderate type: patients with symptoms of fever and signs of respiratory infection, and having pneumonia changes on CT imaging; ③ severe type: patients presenting with any one item of the following: a. respiratory distress, respiratory rate ≥ 30/min; b. oxygen saturation of finger ≤93% in resting condition; c. arterial partial pressure of oxygen (PaO_2_)/oxygen concentration (FiO_2_) ≤ 300 mmHg (1 mmHg = 0.133 kPa); ④ critical type: patients meeting any one of the following criteria: a. respiratory failure requiring mechanical ventilation; b. shock; c. requiring ICU admission requirement due to multiple organ failure [12].

### Statistical analysis

Statistical analysis was performed using software SPSS version 22.0. Continuous variables were expressed as medians and interquartile ranges, or simple ranges, as appropriate. Categorical variables were summarized as counts and percentages.

## Results

### Characteristics of the included patients

Demographics and clinical characteristics are summarized in Table 1. The eight patients included 3 males and 5 females, with ages from 1 to 5 years (median age, 2.5 years [interquartile range, 1.25 to 3.75]). The time from onset to initial diagnosis was 1–10 days, with a median of 7 days (interquartile range, 5.25 to 8.75). Two cases (25%) had a travel history to or from Wuhan, two cases (25%) had family members infected, and four cases (50%) did not have any travel history to or from Wuhan, or of close contact with the patients with laboratory-confirmed COVID-19. Of the eight cases, one (12.5%) had no obvious clinical symptoms. Fever was observed in six cases (62.5%), including one of high fever (> 39 °C), two (12.5%) of moderate fever (37.3–38.0 °C), and three (37.5%) of mild or intermittent fever. There were seven cases of cough, including three (37.5%) of dry cough, five (62.5%) of intermittent mild cough, four cases of productive cough (50%). Three cases (37.5%) showed runny nose. Regarding the severity classification of COVID-19, two cases (25%) were classified as mild type, five cases (62.5%) were classified as moderate type, and one case (12.5%) was classified as critical type and had a coinfection of SARS-CoV-2 and mycoplasma. In addition, one case had a past medical history of surgery for nephroblastoma, and one case had a previous history of surgery for congenital heart disease.

**Table 1:** Demographics and clinical characteristics of 8 hospitalized preschool children infected with COVID-19

Characteristic	Patients							
	1	2	3	4	5	6	7	8
Demographics								
Sex	Male	Female	Male	Female	Female	Female	Male	Female
Age	1y	2 y	3 y	3 y	4 y	5 y	1y	2y
Time from onset to initial diagnosis	5d	7d	10d	8d	7d	6d	9d	1d
Symptoms at onset								
Fever	Yes	Yes	Yes	Yes	No	No	Yes	Yes
Cough	Yes	Yes	Yes	Yes	No	Yes	Yes	Yes
Runny nose	No	Yes	No	Yes	No	No	No	Yes
Epidemiologic history								
Exposure to Wuhan	Yes	No	No	No	No	No	Yes	No
No. of family members infected	0	0	0	0	0	1 (mother)	0	1 (mother)
Laboratory examination								
WBC (10^9^/L)	6.66	9.68	10.75 ↑	4.93	7.12	6.45	8.31	7.28
Neutrophils (10^9^/L)	2.92	3.31	3.85	2.83	3.45	2.97	7.58	3.02
Neutrophils (%)	43.8 ↓	34.3 ↓	35.9 ↓	57.4	33.2 ↓	59.2	83.2 ↑	54.3
Lymphocyte (10^9^/L)	3.15	5.6 ↑	6.22 ↑	1.66	2.12	3.25	1.78	1.97
Lymphocyte (%)	47.4 ↑	57.8 ↑	57.9 ↑	33.6	48.2 ↑	31.8	16.5 ↓	59.2 ↑
Eosinophils (10^9^/L)	0.12	0.14	0.13	0.09	0.08	0.10	0.12	0.17
Eosinophils (%)	1.8	1.4	1.1	1.8	1.3	1.5	1.6	1.4
Basophils (10^9^/L)	0.04	0.02	0.02	0.01	0.05	0.02	0.06	0.01
Basophils (%)	0.6	0.2	0.2	0.3	0.2	0.4	0.4	0.3
ESR (mm/H)	8	12	5	11	7	6	9	12
CRP (mg/L)	0.75	0.5	0.85	32.1 ↑	0.65	1.22	0.89	1.03
Past medical history	Surgery ^a^	no	no	no	no	no	Surgery ^b^	no
Coexisting infection	no	no	no	no	no	no	Mycoplasma pneumonia	no
Clinical type	Moderate	Moderate	Moderate	Moderate	Mild	Mild	Critical	Moderate

↑ indicates an increase and ↓ indicates a decrease in these laboratory examination indexes. ^a^ patient 1 had a past medical history of surgery for nephroblastoma; ^b^ patient 7 had a past medical history of surgery for nephroblastoma congenital heart disease

Abbreviations: *WBC* white blood cell, *ESR* Erythrocyte sedimentation rate, *CRP* C-reactive protein

### Laboratory findings

Routine peripheral blood testing was performed for all cases. One case (12.5%) had increased white blood cell counts, with normal counts in the remaining cases. Four cases (50%) exhibited decreased neutrophil percentages and increased lymphocyte counts and/or percentages. In one case (12.5%), the neutrophil percentage was increased and lymphocyte percentage was decreased. One case (12.5%) exhibited normal neutrophil and increased lymphocyte percentage. One case (12.5%) showed increased C-reactive protein level (Table 1).

### Chest CT imaging findings

**Fig. 1:**
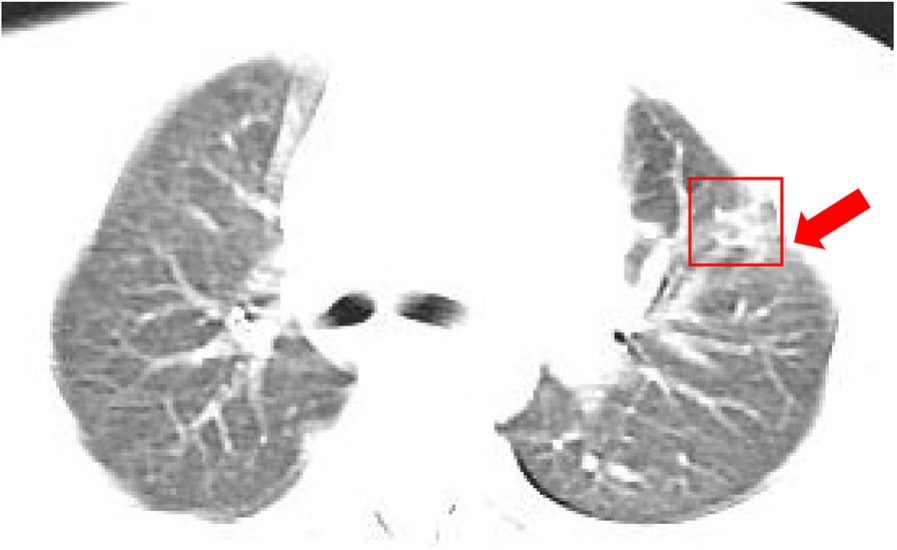
Chest CT images of a 3-year-old male patient show patchy consolidation located in the subpleural region of the right upper lobe (red box), with thickening in the adjacent pleura (red arrow)

**Fig. 2:**
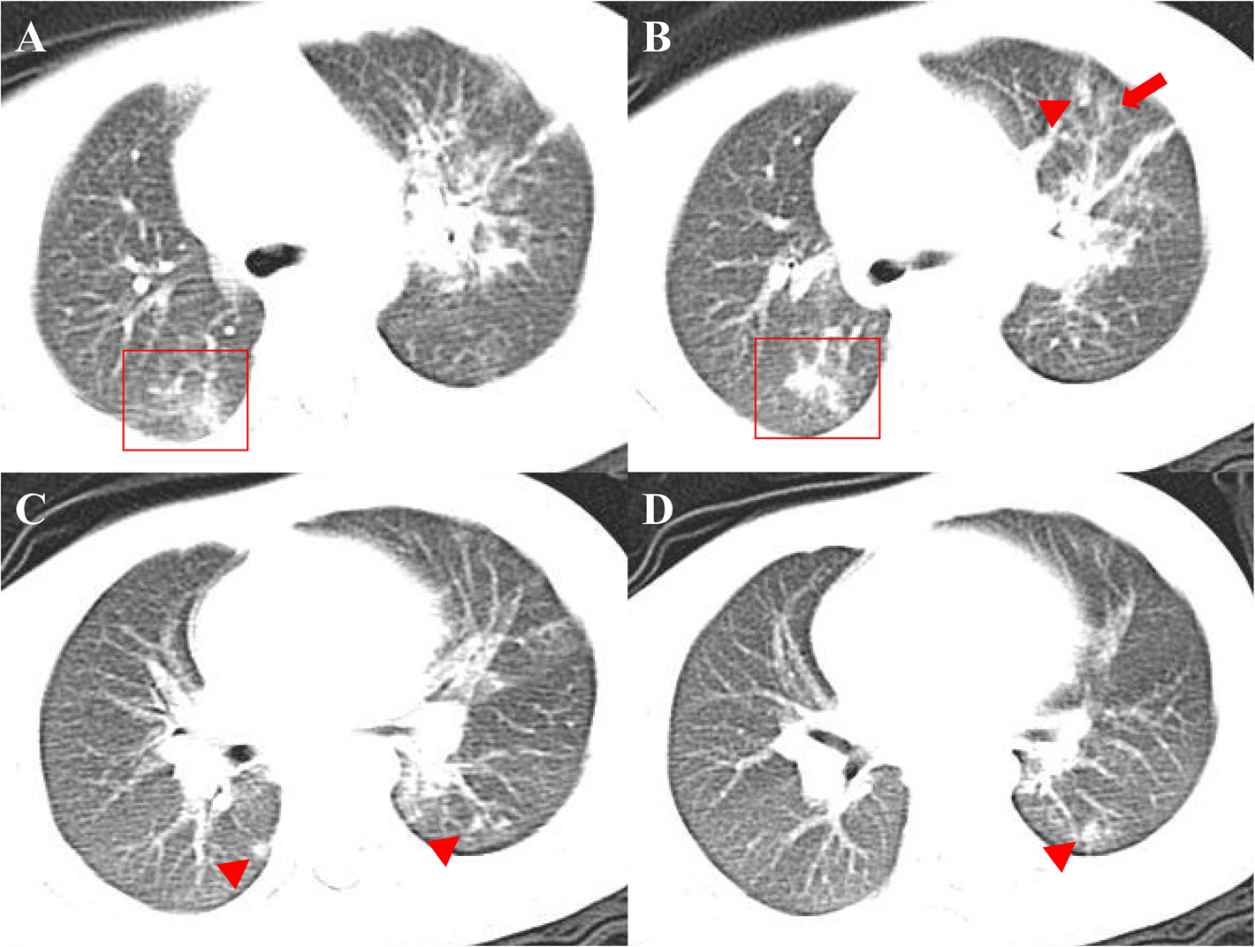
Chest CT images of a 2-year-old female patient show multiple patchy/nodular ground-glass opacity and consolidation, with blurry margins; the patchy/nodular consolidation is mostly located in the mid and inner zones of the both lower lung lobe (A-D, red boxes and triangles), with high density inside the lesions and relatively low density in the periphery, presenting a “halo sign” appearance; the GGO was located in the outer zone of the left upper lobe (B, red arrow)

**Fig. 3:**
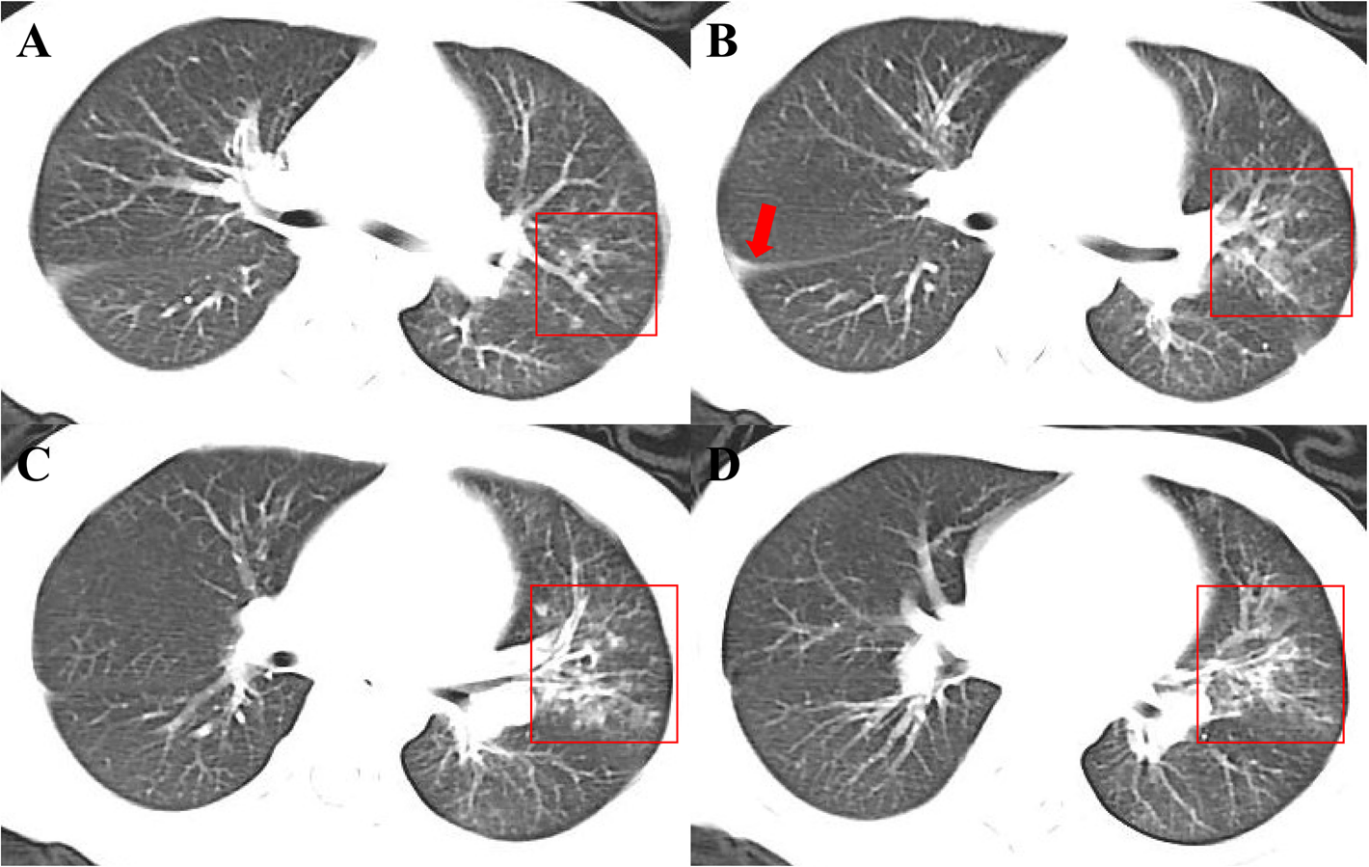
Chest CT images of a 3-year-old female patient show bronchial pneumonia-like changes in the left upper lobe, with multiple high-density nodular shadows and patchy ground-glass opacity along the bronchovascular bundles (A-D, red box). Thickening of the right interlobar pleura is also shown (B, red arrow)

**Fig. 4:**
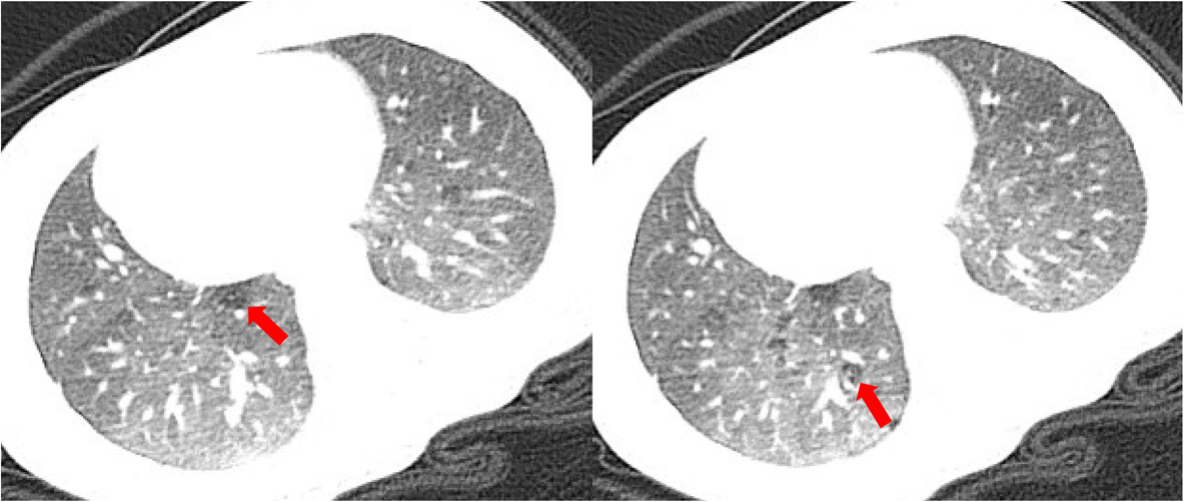
Chest CT images of a 2-year-old female patient show multiple saccular lucency shadows in bilateral lower lobes with unclear margins (red arrows)

**Fig. 5:**
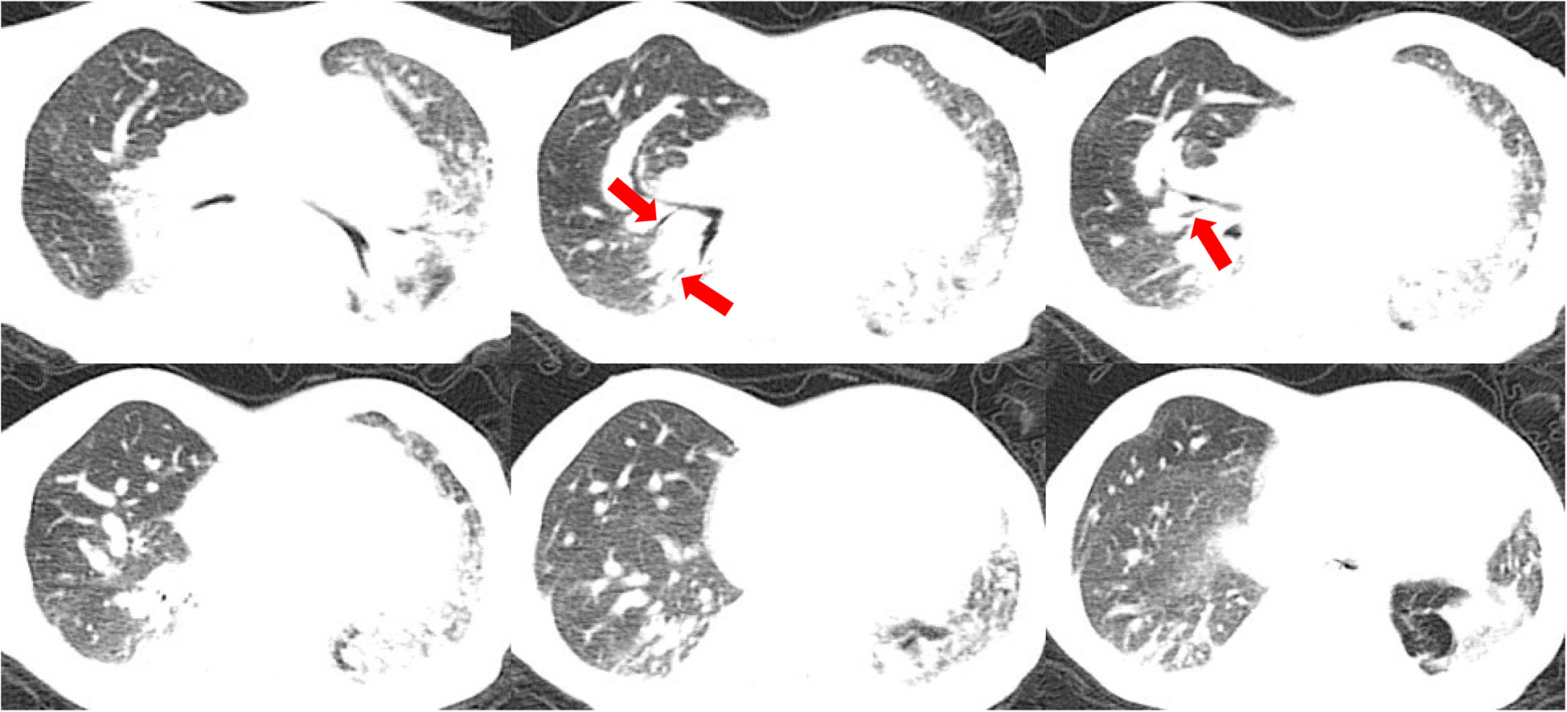
Chest CT images of a 1-year-old male patient with a coinfection of SARS-CoV-2 and mycoplasma show bronchial pneumonia-like changes in both lungs, especially the left lung, presenting blurred and messy bilateral lung markings and multiple patchy shadows scattered along the lung markings with blurry margins. Air bronchogram is also shown (red arrows)

Of the two mild cases, one case did not show positive lung CT findings, and one case only showed minimal pleural thickening on the right side. Among the five moderate cases, one case displayed consolidation located in the subpleural region of the right upper lobe, with thickening in the adjacent pleura (Fig. 1). One case presented multiple consolidation and GGO, with blurry margins; the patchy/nodular consolidation was mostly located in the mid and inner zones of the both lower lung lobe, with high density inside the lesions and relatively low density in the periphery, presenting a “halo sign” appearance; the GGO was located in the outer zone of the left upper lobe (Fig. 2). One case displayed bronchial pneumonia-like changes in the left upper lobe, with multiple high-density nodular shadows and GGO along the bronchovascular bundles (Fig. 3). Two cases displayed asthmatic bronchitis-like changes. One of the two cases showed increased lucency shadows in the right upper lobe, and another case exhibited multiple saccular lucency shadows in the bilateral lower lobes with unclear margins (Fig. 4). In addition, the chest CT examination of the one case who was classified as critical type revealed bronchial pneumonia-like changes in the bilateral lungs, presenting blurred and messy bilateral lung markings and multiple patchy shadows scattered along the lung markings with blurry margins (Fig. 5). There were no pleural effusions, hilar lymphadenopathy, or other signs in this group of patients.

### Treatment and prognosis

Except for the one severe case, the remaining seven mild and moderate cases were routinely administered symptomatic medications. Lian Hua Qing Wen capsules, oseltamivir, and other medications were commonly used. The patient with the shortest disease course had a negative nucleic acid test after 6 days. All patients were followed up for 1 month. After 8–12 days, most of the nucleic acid tests were negative. Most of the patients displayed mild symptoms and favorable outcomes. One case was discharged after treatment according to the discharge criteria outlined in the *Guidelines for the Diagnosis and Treatment of Novel Coronavirus Pneumonia* [11]. By the end of follow-up, six patients had entered the clinical isolation stage, while the severe case was still being treated with ventilator.

## Discussion

Different age group are generally susceptible to SARS-CoV-2. Du et al. estimated that the basic reproduction number (R0 value) is 2.56 [13]. Since December 2019, there have been COVID-19 confirmed cases in all provinces and cities nationwide in China, and in many other countries. As of 6 February 2020, at least 230 COVID-19 cases in children (≤18 years) have been reported in China [14], suggesting that the SARS-CoV-2 has a strong transmission capacity in the special population (i.e., neonate and children). The present study indicates that infected preschool children have different clinical symptoms and CT imaging findings from those of adults. Symptoms may not be apparent in young children. Under the current epidemic conditions, cluster outbreaks in families have occurred. If children, especially preschool children, are not paid attention to, they may contribute to the spread of infection and may not be treated in a timely manner.

The incubation period of the novel coronavirus infection in children can be as short as 1 day or as long as 14 days, with an estimated mean of 5.2 day s[15]. Of the eight cases in this study, two subjects had a travel history to Wuhan for surgery; however, these two cases were in Wuhan prior to the outbreak of the COVID-19. Based on the history of exposure, their incubation periods were greater than 14 days. Thus, it is reasonable to think that the COVID-19 already existed in Wuhan before January 2020, or that the two cases were all postoperative cases with low immunity who were infected with the SARS-CoV-2 after they returned home. Findings from a recent study also indicate that SARS-CoV-2 infections in children were occurring early in the epidemic [16]. Of the six remaining cases, two mild cases had a history of family members (mothers) infected, and thus were familial outbreaks. In four cases, the parents denied or were uncertain concerning the history of contact with the epidemic area.

Epidemiologically, viral pneumonia is an inhalation infection that is directly transmitted from human-to-human through sputum and droplets. The infection is mainly caused by pulmonary lesions due to the downward spread of upper respiratory virus. Since preschool children tend not to actively enter high-traffic places, the possibility of transmission through droplets is markedly lower than that in adults. The infection may be “second-generation” or “third-generation”. Therefore, epidemiology is important in the diagnosis of COVID-19 in preschool children, although the final diagnosis remains dependent on the nucleic acid test.

Our study revealed that the clinical manifestations of COVID-19 in preschool children are different from those in adults. The main clinical manifestations of COVID-19 in adults are fever and cough [7]. Of the eight cases in our study, only one exhibited high fever. Clinical symptoms of COVID-19 in preschool children are relatively mild, and most are diagnosed as mild or moderate. This may be related to the non-susceptible physiological basis of children. The prevalence of severe acute respiratory syndrome in children is also lower than that in adults. Considering the common characteristics of coronaviruses, children may be relatively less susceptible based on their cellular structure or immunity.

The present findings indicate that the chest CT features of COVID-19 pneumonia in preschool children differ from those in adults. The early-stage imaging features of preschool children are not typical, mainly presenting consolidations, while the most common early-stage CT feature in adult patients with COVID-19 pneumonia is the GGO distributed along the bronchovascular bundle or the dorsolateral and subpleural part of the lungs [12]. Pulmonary consolidations were smaller in the present cases and showed changes that included small patches or nodules with unclear margins. There were no pure GGO, or “crazy paving sign”, or other imaging features that are typical findings in adult patients at the early stage [17–19]. The chest CT manifestations of the eight cases were mostly mild or moderate, with better outcomes after treatment. The lesions were significantly absorbed in 6–12 days. This is different from changes in adult that pulmonary interstitial fibrosis is common after treatment. In our study, there was one critical case who was only 8 months old and had a previous history of surgery and combined with mycoplasma infection, suggesting that infants can also be infected with the SARS-CoV-2, and that a previous surgical history and comorbidity may aggravate the progression of COVID-19. Asthmatic bronchitis- and bronchial pneumonia-like changes are also characteristics of COVID-19 pneumonia in preschool children, which were found in four cases in our study. In addition, the COVID-19 pneumonia in preschool children can manifest as small airway lesions, with uneven lucency of the regional lung lobe or multiple small cystic lucency shadows in the bilateral lower lungs.

The epidemic of COVID-19 began during the winter season in China. Common diseases seen in young children in winter are mycoplasma pneumonia, influenza A (H1N1) with pneumonia, and adenovirus pneumonia. Therefore, the CT features of COVID-19 pneumonia need to be differentiated from those infectious diseases. The most common CT manifestations of mycoplasma pneumonia are bilateral peribronchial perivascular interstitial infiltrations in central and middle lung zones [20]. One case in our study was confirmed to have mycoplasma and SARS-CoV-2 infection at the same time. Adenoviral pneumonia mostly occurs in children, mainly involving the middle and inner zones of bilateral lungs and presenting hilar enlargement, pleural effusion, pneumothorax, mediastinal emphysema, subcutaneous emphysema, while involvement of subpleural areas is rare [21]. The most common imaging features of H1N1 pneumonia are unilateral or bilateral GGO with or without associated focal or multifocal areas of consolidation, and the GGO and areas of consolidation had a predominant peribronchovascular and subpleural distribution [22, 23], which is difficult to distinguish from CT findings of COVID-19 pneumonia.

We acknowledge several limitations in this study. First, it was a retrospective study with a small sample size. Second, a co-infectious case (SARS-CoV-2 and mycoplasma pneumoniae) was included in this study, which might give confusion for the CT findings of COVID-19 pneumonia.

## Conclusion

In summary, the chest CT imaging features and clinical manifestations of COVID-19 in preschool children are atypical and various, and are relatively mild or moderate compared with adult patients. Familial aggregation infection may be the main cause of COVID-19 in preschool children. It is difficult to distinguish the CT manifestations of COVID-19 pneumonia from those of other common pneumonia occurring in winter. Finding of the present study may help accelerate our understanding of the CT manifestations of COVID-19 pneumonia, thus can make up for the false negative results of RT-PCR testing and be helpful for early isolation and intervention of SARS-CoV-2 infected children. Accurate diagnosis of COVID-19 still needs a comprehensive evaluation of epidemiological, clinical, laboratory and CT imaging data for the special population.

## Data Availability

The datasets used and analyzed during the current study are available from the corresponding author on reasonable request.

## Abbreviations

GGO: Ground-glass opacity
CT: Computed tomography
COVID-19: Coronavirus disease 2019
SARS-CoV-2: Severe acute respiratory syndrome coronavirus 2
RT-PCR: Reverse transcription polymerase chain reaction
WHO: World Health Organization
